# Public Health and Climate Benefits and Trade‐Offs of U.S. Vehicle Electrification

**DOI:** 10.1029/2020GH000275

**Published:** 2020-10-01

**Authors:** D. R. Peters, J. L. Schnell, P. L. Kinney, V. Naik, D. E. Horton

**Affiliations:** ^1^ Program in Environmental Sciences Northwestern University Evanston IL USA; ^2^ Environmental Defense Fund Austin TX USA; ^3^ Department of Earth and Planetary Sciences and Institute for Sustainability and Energy Northwestern University Evanston IL USA; ^4^ Cooperative Institute for Research in Environmental Sciences University of Colorado Boulder NOAA/Global Systems Laboratory Boulder CO USA; ^5^ Department of Environmental Health Boston University School of Public Health Boston MA USA; ^6^ NOAA Geophysical Fluid Dynamics Laboratory Princeton NJ USA

**Keywords:** health impact analysis, electric vehicles, air quality, climate change, co‐benefits

## Abstract

Vehicle electrification is a common climate change mitigation strategy, with policymakers invoking co‐beneficial reductions in carbon dioxide (CO_2_) and air pollutant emissions. However, while previous studies of U.S. electric vehicle (EV) adoption consistently predict CO_2_ mitigation benefits, air quality outcomes are equivocal and depend on policies assessed and experimental parameters. We analyze climate and health co‐benefits and trade‐offs of six U.S. EV adoption scenarios: 25% or 75% replacement of conventional internal combustion engine vehicles, each under three different EV‐charging energy generation scenarios. We transfer emissions from tailpipe to power generation plant, simulate interactions of atmospheric chemistry and meteorology using the GFDL‐AM4 chemistry climate model, and assess health consequences and uncertainties using the U.S. Environmental Protection Agency Benefits Mapping Analysis Program Community Edition (BenMAP‐CE). We find that 25% U.S. EV adoption, with added energy demand sourced from the present‐day electric grid, annually results in a ~242 M ton reduction in CO_2_ emissions, 437 deaths avoided due to PM_2.5_ reductions (95% CI: 295, 578), and 98 deaths avoided due to lesser ozone formation (95% CI: 33, 162). Despite some regions experiencing adverse health outcomes, ~$16.8B in damages avoided are predicted. Peak CO_2_ reductions and health benefits occur with 75% EV adoption and increased emission‐free energy sources (~$70B in damages avoided). When charging‐electricity from aggressive EV adoption is combustion‐only, adverse health outcomes increase substantially, highlighting the importance of low‐to‐zero emission power generation for greater realization of health co‐benefits. Our results provide a more nuanced understanding of the transportation sector's climate change mitigation‐health impact relationship.

## Key Points

- Electrifying vehicles consistently reduces carbon dioxide emissions; air quality benefits vary spatially and depend on charging energy mix
- Twenty‐five percent U.S. electric vehicle adoption with energy sourced from current electric grid prevents ~$17B in health and climate damages annually
- Peak co‐benefits occur with greater fractions of EV adoption and emission‐free energy sources

## 1 Introduction

Emission and accumulation of greenhouse gases (GHGs) in Earth's atmosphere has increased radiative forcing, led to global climatic change, and motivated mitigation and adaptation planning (Intergovernmental Panel on Climate Change, 2018; Myhre et al., 2014). Among the most compelling GHG reduction measures proposed are those with economic, social, and/or health co‐benefits (Haines, 2017; Patz et al., 2020; Rogelj et al., 2016; Thompson et al., 2014). Co‐beneficial actions include relatively facile measures, such as the promotion of active transport and reduced meat consumption, as well as more high‐inertia efforts including preservation and expansion of urban greenspaces and the transformation of carbon‐intense transportation and energy infrastructure. Evidence suggests that quantifying co‐beneficial outcomes in climate change mitigation policy analyses directly addresses concerns of political leaders—primarily that CO_2_ mitigation is costly and has limited local benefits (Granoff et al., 2016; Nemet et al., 2010). Indeed, previous efforts seeking to elucidate the ancillary benefits of GHG reduction scenarios have demonstrated that the economic benefits from concomitant reductions in air pollutants and their attendant health impacts can exceed the costs of GHG abatement (West et al., 2013). Here, we present a transportation sector‐targeted analysis of the co‐benefits and trade‐offs of the electrification of light‐duty passenger vehicles (LDPV) in the United States. EV adoption has the potential to provide concomitant reductions in air pollutants and GHGs thereby providing both positive (and often local) health and economic benefits.

We target the U.S. light‐duty transportation sector for several reasons. In 2017, U.S. transportation sector GHG emissions surpassed all other individual sectors, accounting for 29% of the country's total GHG emissions. Within the transportation sector, ~60% of GHG emissions came from light‐duty vehicles (U.S. Environmental Protection Agency [EPA], 2019a). As governments at the state‐, county‐, and city‐level develop Climate Action Plans (CAPs) to reduce GHG contributions, transportation GHG reductions have been a key focus (City of Chicago, 2008; Los Angeles County Department of Regional Planning, 2015; New York City Mayor's Office of Sustainability, 2017). Additionally, nine states have identified light‐duty passenger vehicles as their single largest GHG emissions source and implemented an action plan to accelerate electric vehicle adoption through strategies including infrastructure investment and consumer incentives (Multi‐State Zero Emission Vehicle (ZEV) Task Force, 2018).

In addition to being a leading contributor to GHG emissions, the U.S. transportation sector is responsible for air pollutant emissions that cause a substantial public health burden. Light‐duty vehicle emissions include primary and secondary pollutants that comprise or contribute to atmospheric fine particulate matter (PM_2.5_) and ground‐level ozone (O_3_)—both of which are criteria air pollutants with well‐documented human health impacts (U.S. EPA, 2018a). Fann et al. (2013) estimate that ground‐level O_3_ and PM_2.5_ from mobile source emissions cause between 19,300 and 54,000 premature deaths per year in the United States. Similarly, a recent International Council on Clean Transportation report estimated that the United States experienced 22,000 transportation‐attributable ambient PM_2.5_ and O_3_ deaths in 2015 (Anenberg et al., 2019). Most recently, Davidson et al. (2020) estimated a health burden of 12,000–31,000 premature deaths in the United States for on‐road emissions alone in the year 2011. Given the magnitude of the health burden associated with ambient air pollution from traffic, reducing vehicle emissions through vehicle electrification is a clear opportunity for mitigating air pollution‐related health effects while also reducing climate forcing from CO_2_ and short‐lived climate pollutant emissions. Indeed, the aforementioned CAPs and ZEV Action Plan all cite potential air quality co‐benefits of reduced vehicle emissions. Therefore, a comprehensive analysis of climate and health co‐benefits of transportation electrification scenarios is needed to ensure that EV policy initiatives achieve optimal intended outcomes, particularly when past studies have demonstrated that EV‐derived health benefits are not as clear‐cut as CO_2_ reductions (Requia et al., 2018; Tessum et al., 2014, and references therein).

Prior analyses of the emission changes associated with vehicle electrification have primarily focused on GHG emission reductions from a climate change mitigation perspective (e.g., Requia et al., 2018; Richardson, 2013). Studies have shown that GHG emissions decrease with LDPV electrification even when the power source for battery charging is coal‐fired power plants—due to their higher power generation efficiencies in comparison with distributed gasoline‐powered internal combustion engines (Huo et al., 2015; Requia et al., 2018). In contrast, the limited existing literature on the air quality impacts of EV adoption has shown a greater dependency on the battery charging energy generation source (Requia et al., 2018). Two China‐based EV adoption studies found conflicting results—Huo et al. (2013) found that the total internal combustion engine fleet replacement with EVs would increase PM emissions, while Liang et al. (2019) found that the air quality benefits from EV adoption would avoid over 17,000 deaths annually in addition to reducing GHGs. U.S.‐based studies also find nuanced air quality impact differences from EV adoption. Nopmongcol et al. (2017) found that electrification of 17% of light‐duty vehicles could lead to modest but widespread reductions in O_3_ and particulate matter, whereas Schnell et al. (2019) demonstrated that for PM_2.5_ in particular, EV adoption benefits varied by region and season, and depended largely on the power generation mix used for marginal EV charging. Indeed, Tessum et al. (2014) found that in the United States, the health outcome of a 10% EV adoption depended heavily on the type of energy used to charge the EVs. These results reflect a complex trade‐off between transportation emissions and power generation emissions and suggest that the regional energy generation mix used to charge EVs heavily influences air quality and health outcomes.

In this study, we focus on premature mortality as a health endpoint, as it captures a range of cardiovascular and respiratory pathways. We use a suite of health impact functions (HIFs) to assess the epidemiological uncertainties from exposure to PM_2.5_ and O_3_ (Bell et al., 2004; Ito et al., 2005; Jerrett et al., 2009; Krewski et al., 2009; Laden et al., 2006). To elucidate the co‐benefits and trade‐offs of EV adoption, we use CO_2_ emission data and chemistry‐climate model simulated surface concentrations of O_3_ and PM_2.5_ from two different U.S. EV fractional adoption scenarios under three different battery charging power generation configurations. To provide a more comprehensive understanding of the distribution of U.S. health and climate co‐benefits under potential future vehicle and energy scenarios, we analyze public health and CO_2_ emission benefits and trade‐offs for individual states and geographic regions.

## 2 Data and Methods

### 2.1 Air Quality Simulations and Emissions Scenarios

EV adoption scenarios were developed for coupled atmospheric chemistry and climate model sensitivity simulations by Schnell et al. (2019). We use the model‐simulated hourly surface pollutant (O_3_ and PM_2.5_) abundances from Schnell et al.'s six hypothetical EV adoption scenarios (Table 1), as well as the baseline control run. Two different EV adoption proportions were considered under three different energy generation regimes. Conventional LDPV proportions of 25% and 75% were instantaneously replaced with battery powered electric vehicles. To produce the additional electricity to charge EV batteries, varying levels of emission‐free power generation sources (wind, solar, hydro, and nuclear) were considered: that is, *r0* (no emission‐free sources, i.e., all combustion sources), *rC* (a state's current grid mix), and *r2C* (doubles each state's fraction of emission‐free generation sources). For *rC* and *r2C*, the emission‐free power generation fraction is based on an individual state's current emission‐free generation capacity, that is, any state whose current energy generation is composed of less than 50% emission‐free power, will produce a fraction of its needed electricity from combustion sources in the *r2C* scenario. When referring to these scenarios throughout the paper, we use the notation *eX‐rY*, where *X* is the percentage of LDPVs converted to EVs and *Y* indicates the proportion of energy coming from emission‐free grid sources (Table 1).

**Table 1:** Modeled EV Scenarios

Scenario	% EV conversion	Energy generation infrastructure for EV charging
*BASE*	0	2014 mix
*e25‐r0*	25	2014 mix with no emission‐free sources
*e25‐rC*	25	2014 mix
*e25‐r2C*	25	2014 mix with doubled fraction of emission‐free sources
*e75‐r0*	75	2014 mix with no emission‐free sources
*e75‐rC*	75	2014 mix
*e75‐r2C*	75	2014 mix with doubled fraction of emission‐free sources

*Note*. Six EV adoption scenarios are considered, in addition to a baseline simulation. EV replacement of 25% and 75% of the U.S. light‐duty passenger vehicle fleet was simulated under three different energy generation configurations. That is, the state‐level fraction of energy required to charge EV batteries is sourced from *r0* (no emission‐free sources), *rC* (current grid mix), and *r2C* (doubles each state's fraction of emission‐free generation sources).

Emission changes (Δ*E*) resulting from EV adoption were calculated as

(1) $$\Delta E=-E^{\text{LDPV}}+E^{\mathrm{EGU}}$$

where *E*^*LDVP*^ are the removed emissions (see Table 2) of LDPVs and *E*^*EGU*^ are the added emissions from combustion‐fired electric generating units (EGUs). LDPV emissions for 2014 are obtained from U.S. Environmental Protection Agency (EPA) National Emissions Inventory 2014 (U.S. EPA, 2014), and power plant emissions are obtained by multiplying remapped electricity demand required by the newly placed EVs by e‐GRID reported power plant emission rates (U.S. EPA, 2017). e‐GRID is also used for state‐level renewable energy fraction, which we assume is uniform across each state (Schnell et al., 2019). We assume that the adopted EVs have an efficiency of 0.16 kWh km^−1^ (similar to a 2020 Nissan Leaf or Tesla Model 3 Standard). The complete methods for modifying the emissions for the EV scenarios can be found in Schnell et al. (2019). National emission changes by EV scenario are shown in Table 2.

**Table 2:** Simulated Latitudinally Weighted National Average Emission Changes for EV Adoption Scenarios

	EV adoption scenarios					
Emission Δ (Gg)	*e25‐r0*	*e25‐rC*	*e25‐r2C*	*e75‐r0*	*e75‐rC*	*e75‐r2C*
NO	−183.5	−228.2	−268.2	−550.5	−684.6	−804.4
SO_2_	282.6	202.6	127.1	847.7	608	381.2
OM	0.3	−1	−2.1	0.7	−3.2	−6.2
BC	−1.1	−1.5	−1.8	−3.5	−4.5	−5.4
CO	−3,366	−3,394	−3,416	−10,099	−10,181	−10,247
C_4_H_10_	−6.4	−42.3	−76.4	−19.2	−126.7	−229.2

*Note*. Changes (in gigagrams) are computed relative to the *BASE* simulation for each scenario. Percent changes by U.S. region are also available in Table S4 of Schnell et al. (2019).

Emissions developed for the six scenarios were used to drive global simulations of a prototype version of the Geophysical Fluid Dynamics Laboratory Atmospheric model version 4 (GFDL‐AM4) model at 0.5° resolution for the year 2014, following a 1‐year spin‐up. In our simulations, the GFDL‐AM4 atmospheric chemistry‐climate model utilizes observed sea surface temperature and sea ice distribution boundary conditions. Simulations use a 30‐min time step and are nudged to 2014 NCEP reanalysis winds to facilitate direct comparison to air quality observations (see Figures S1–S3 in Schnell et al., 2019). The model includes detailed tropospheric and stratospheric gas‐phase chemistry and simulates the major components of fine particulate matter (PM) including hydrophilic and hydrophobic black carbon and organic matter, ammonium, sulfate, nitrate, sea salt, mineral dust, and secondary organic aerosols (SOA). Sea salt, mineral dust, and biogenic emissions of isoprene and monoterpenes are calculated interactively. Biogenic SOA is emitted as a 5% yield of isoprene and monoterpene emissions, and anthropogenic SOA is formed by a 5% yield of the oxidation of the lumped n‐butane species by OH. Our baseline scenario (*BASE*) employs Coupled Model Intercomparison Project Phase 6 (CMIP6) emissions (Hoesly et al., 2017) to include surface anthropogenic, biomass burning, and aircraft components. The *BASE* scenario was evaluated in Section 3.1 “Model evaluation” and Figures S1–S3 of Schnell et al. (2019), which show seasonal biases and correlations between modeled and observed O_3_ and PM_2.5_ concentrations across the United States. In short, simulated O_3_ concentrations were biased high, while PM_2.5_ concentrations were biased low, except in the Southeast.

From the hourly model‐calculated surface O_3_ (ppb) and PM_2.5_ (μg m^−3^) abundances over the contiguous United States, we calculate the maximum daily 8‐hr average (MDA8) for O_3_ and the 24‐hr average for PM_2.5_, which serve as inputs for our health impact analyses. To complement the air quality changes and determine co‐benefits, for each scenario we compute CO_2_ emission changes using the Schnell et al. (2019) remapping algorithm (Equation 1). For example, in the *e25‐rC* scenario, *E*^*LDVP*^ is 25% of the total 1.2 Gt U.S. LDPV CO_2_ emissions for 2014 and *E*^*EGU*^ is the added CO_2_ emissions from EGUs.

### 2.2 Health Impact Calculations

We use the U.S. EPA Environmental Benefits Mapping and Analysis Program‐Community Edition v1.5 (BenMAP‐CE; U.S. EPA, 2019b) to analyze changes in premature mortality resulting from changes in O_3_ and PM_2.5_ in each electrification scenario. BenMAP‐CE calculates changes in adverse health effects using population data, baseline rates of incidence and prevalence of disease, and HIFs from epidemiological studies that quantify associations between health endpoints and changes in pollutant concentrations. The BenMAP‐CE baseline mortality data are derived from 2012 to 2014 Center for Disease Control and Prevention's WONDER database, which projects 5‐year intervals using annual adjustment factors based on U.S. Census Bureau projected national mortality rates (U.S. EPA, 2018b). Population information is derived from 2010 U.S. Census block‐level data projected to 2050 using economic growth factors (Voorhees et al., 2011). Selected HIFs follow a log‐linear relationship (Equation 2) to calculate the change in adverse health effects (Δ*y*, deaths avoided year^−1^) for each grid cell, where *y*_0_ represents the baseline incidence rate of the adverse health effect, Δ*AQ* represents the change in pollutant concentration, *Pop* is the population exposed, and *β* is a coefficient derived from the concentration‐response function of a given epidemiological study that estimates the response of a health outcome to a change in pollutant concentration (Sacks et al., 2018).

(2) $$\Delta y=y_{0}\times \left(e^{\beta \Delta \mathrm{AQ}}-1\right)\times \mathrm{Pop}$$

Most of the results we present utilize HIFs from two widely cited epidemiological studies: Krewski et al. (2009) for PM_2.5_ and Bell et al. (2004) for O_3_ (Table S1). Following recommended best practices, we also assess the sensitivity of our findings to the chosen HIFs by considering two additional functions: Laden et al. (2006) for PM_2.5_ and Ito et al. (2005) for O_3_. These additional HIFs quantify relationships between pollutant concentration and health endpoints similar to Krewski et al. and Bell et al. but differ in that they are derived from different cohorts (i.e., different populations, locations, times, and environmental conditions). A large body of evidence in the epidemiological literature links O_3_ exposure to short‐term mortality (Fann et al., 2012), which both Bell et al. (2004) and Ito et al. (2005) quantify; however two recent studies identified associations between O_3_ exposure and long‐term mortality (Jerrett et al., 2009; Turner et al., 2016). We therefore also include O_3_ HIF results from a third study, Jerrett et al. (2009), to consider the sensitivity of our results to long‐term O_3_ exposure health impacts. We focus the majority of our health impact analyses on results that use the Krewski et al. and Bell et al. methods, as they represent the most conservative damage estimates of the HIFs employed. Table S1 details the properties of each HIF, including study locations, age groups, health endpoints, and timeframe (i.e., long‐term vs. short‐term). The two PM_2.5_ HIFs and the long‐term O_3_ HIF apply only to adult populations (Krewski et al. & Jerrett et al., 30–99; Laden et al., 25–99), and thus the health outcomes we quantify using these functions are limited to these population fractions and are not representative of the total population burden.

Pollutant data from our model simulations were input into BenMAP‐CE using the appropriate metric for each HIF for each pollutant (i.e., daily mean for PM_2.5_, MDA8 for O_3_). The change in concentration of each air pollutant is the difference between the *BASE* simulation and electrification scenario simulations at each cell in the model grid. The PM_2.5_ HIFs and the long‐term O_3_ HIF quantify annual health endpoints from an annual average pollution metric. Short‐term O_3_ HIFs quantify aggregated annual health benefits based on daily 8‐hr maximum O_3_ values during the warm season. We use the full concentration range of PM_2.5_ and O_3_ and do not apply a minimum concentration threshold when using the HIFs.

To calculate national statistics, we aggregated grid cell results. Regional statistics were calculated using state‐aggregated health results, which were then divided into four U.S. regions (Figures 3a and 3g) based on the U.S. Census Bureau's classification system (U.S. Census Bureau, 2018). Regions vary in population size and demographics, baseline incidence rates, electricity generation sources, and corresponding emissions—all of which influence the outcome of the health impact analyses.

The uncertainty associated with the *β* coefficient of each HIF utilized is included in the *β* Standard Error column of Table S1, and this uncertainty is incorporated in health impact calculations using Monte Carlo simulations with Latin Hypercube sampling to produce estimates of outcomes at a range of percentiles based on the uncertainty in the health impact function coefficients (Davidson et al., 2007). We report the 2.5% and 97.5% estimates to capture the 95% confidence interval (CI).

To provide an equal‐footing comparison of simulated changes in tonnage of CO_2_ emissions and premature deaths avoided, we calculate the monetary value of each change using derived damage metrics, that is, the U.S. social cost of carbon (SCC; Ricke et al., 2018) and the value of statistical life (VSL; U.S. EPA, 2015; Anenberg et al., 2019). For the social cost of carbon in the U.S., we follow the guidance of Ricke et al. (2018), who use climate model projections, economic damage estimations, and socioeconomic projections to value the expected economic damages from CO_2_ emissions. They find that U.S.‐level carbon emissions are valued at $48 (66% CI: 1, 118) per ton. To estimate the monetary value of health outcomes we follow the methods employed in the International Council on Clean Transportation report on the global impacts of transportation pollution, that is, the value of statistical life in the United States is found to be $9.6 M using labor market estimates (Anenberg et al., 2019; Viscusi & Masterman, 2017). One caveat to bear in mind when considering these estimates is that our valuation estimates assume that benefits from O_3_ and PM_2.5_ reductions, while calculated in isolation, can be added together, thereby ignoring potential interactions between coincident reductions of each pollutant.

## 3 Results

### 3.1 Pollutant Change Summary

**Figure 1:**
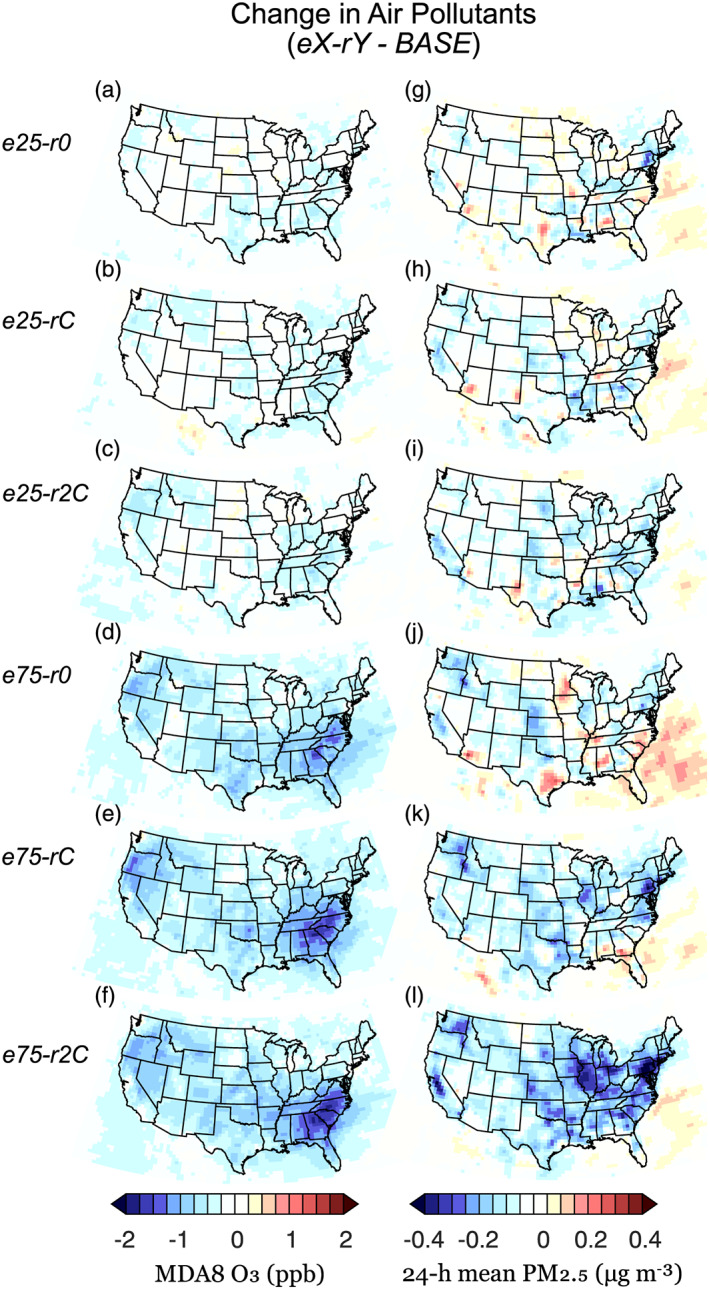
Air pollutant changes. Simulated annual average changes from the baseline scenario for (a–f) O_3_ (MD8A: maximum daily 8‐hr average) and (g–l) PM_2.5_ (24‐hr mean) for each EV adoption‐energy generation scenario.

In aggregate, annual average decreases in national ambient concentrations of O_3_ and PM_2.5_ are simulated under all EV adoption‐energy generation scenarios (Figure 1). Pollutant concentration changes are generally proportional to the EV adoption proportion considered, that is, compare magnitudes of *e25* changes to *e75* changes. Ozone decreases are found to be largely pervasive across scenarios and states (Figures 1a–1f), whereas changes in PM_2.5_ are heterogeneous (Figures 1g–1l). In all simulations, O_3_ decreases are largest in the southeastern United States. PM_2.5_ increases tend to be localized and are prevalent in regions that rely heavily on combustion power generation facilities. These changes are particularly apparent in combustion‐only power generation scenarios (*r0*). In addition to spatial heterogeneities, Schnell et al. (2019) found heterogeneous seasonal pollutant changes (not shown). For example, during the winter, PM_2.5_ decreased throughout the United States in all scenarios due to decreases in nitrate aerosols, whereas increases in PM_2.5_ were primarily simulated in the summer and spring due to increases in more thermally stable sulfate aerosols. Seasonal O_3_ changes were muted in magnitude, with the exception of the summer when simulated changes were an order of magnitude greater than those simulated in the spring, autumn, and winter.

In the remainder of this manuscript, we focus on annualized health metrics that are calculated from annual data (PM_2.5_ and long‐term O_3_ HIFs) and daily warm season O_3_ data (short‐term O_3_ HIFs). However, spatiotemporal air pollutant variations, extreme events, and changes in exposure potential due to climatic change are also critical for better understanding human exposure and public health outcomes of near‐surface pollutant accumulation (Callahan et al., 2019; Gao et al., 2015; Horton et al., 2014). For a comprehensive discussion of the seasonal/regional air pollutant changes and the underlying atmospheric chemistry of the EV adoption scenarios considered here, please consult Schnell et al. (2019).

### 3.2 Aggregated National Climate and Health Co‐benefits

**Figure 2:**
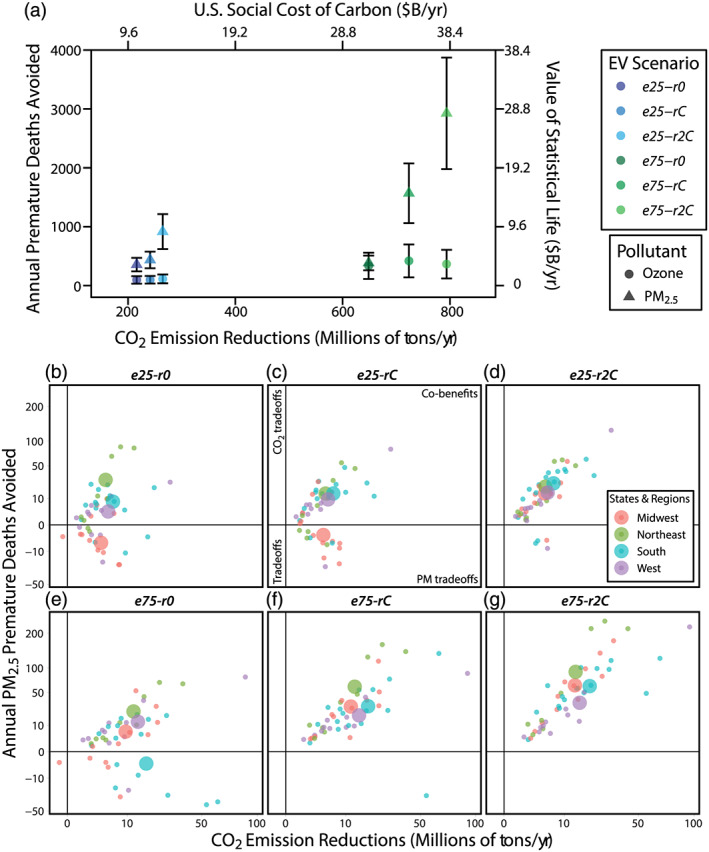
National, regional, and state co‐benefits. Avoided premature mortality and CO_2_ reduction co‐benefits under six vehicle electrification scenarios. (a) National aggregate benefits of CO_2_, O_3_, and PM_2.5_ reductions. Metrics provided include premature deaths avoided, value of statistical life (Anenberg et al., 2019), tonnage of CO_2_ emissions avoided, and the U.S. social cost of carbon ($48 ton^−1^; Ricke et al., 2018). Error bars show the 95% CI for health impact results. Circles indicate premature death avoided from changes in O_3_, as calculated using the Bell et al. (2004) HIF. Triangles indicate PM_2.5_ premature deaths avoided annually, using Krewski et al. (2009). (b–g) Climate and PM_2.5_ health co‐benefits and trade‐offs (Krewski et al., 2009) for individual states (smaller circles) and regional averages (larger circles). For population normalized data see Figure S3.

Regardless of the EV adoption scenario considered, we find that the United States would achieve aggregate national CO_2_ mitigation and mortality reduction benefits (Figure 2a). Aggregate CO_2_ mitigation estimates range from 217 Mt year^−1^ under the least ambitious (*e25‐r0*) adoption scenario wherein EV battery charging is powered by combustion‐only sources, to 796 Mt year^−1^ under the most aggressive adoption‐mitigation scenario (*e75‐r2C*) wherein individual state's renewable energy generation capacity is doubled (Table 3). EV adoption under the current (2014) energy generation mix leads to reductions ranging from 242 to 725 Mt year^−1^ depending on the fraction of EVs that replace ICEs (i.e., *e25‐rC* vs. *e75‐rC*).

**Table 3:** Aggregate Avoided CO_2_ Emissions and Premature Deaths

	***e25‐r0***	***e25‐rC***	***e25‐r2C***	***e75‐r0***	***e75‐rC***	***e75‐r2C***
	**CO**_**2**_ **reduction (10** ^**6**^ **tons per year)**					
	217	242	265	650	725	796
**PM**_**2.5**_ **HIF**	**PM**_**2.5**_ **deaths avoided per year**					
***Krewski et al*.**	**358 (242, 473)**	**437 (295, 578)**	**922** **(623, 1,219)**	**386** **(261, 511)**	**1,576 (1,065, 2086)**	**2,939 (1985, 3,888)**
*Laden et al*.	919 (412, 1,423)	1,122 (503, 1737)	2,369 (1,062, 3,669)	991 (445, 1,534)	4,051 (1817, 6,273)	7,548 (3,386, 11,684)
**O**_**3**_ **HIF**	**Ozone deaths avoided per year**					
***Bell et al*.**	**96 (32, 159)**	**98 (33, 162)**	**113 (38, 188)**	**336 (112, 558)**	**420 (139, 698)**	**366 (121, 608)**
*Ito et al*.	193 (131, 255)	198 (134, 261)	229 (155, 302)	682 (460, 902)	853 (576, 1,128)	742 (501, 982)
*Jerrett et al*.	181 (61, 301)	188 (63, 313)	220 (74, 366)	636 (214, 1,055)	794 (267, 1,316)	702 (236, 1,164)

*Note*. For each EV adoption and energy generation mix scenario (*eX‐rY*), aggregated U.S. CO_2_ emission reductions and avoided premature deaths are computed. Premature death avoided values represent the median and (95% CI) departure from baseline conditions (*BASE*) from a suite of BenMAP‐CE HIFs. The bolded HIFs and values are those which we predominantly discuss in the results and discussion.

Similar to CO_2_, reductions in ambient O_3_ and PM_2.5_ concentrations also provide aggregate U.S. benefits for all EV adoption scenarios considered. Air quality improvements due to EV adoption lead to national aggregate decreases in premature mortality for all charging options (Figure 2a and Table 3). Reduced PM_2.5_ concentrations decrease annual premature deaths by 358 (95% CI: 242, 473) in *e25‐r0* to 2,939 (95% CI: 1985, 3,888) in the *e75‐r2C* scenario. Ozone‐related premature mortality is likewise reduced in these scenarios, with changes ranging from 96 (95% CI: 32, 159) to 366 (95% CI: 121, 608) in *e25‐r0* and *e75‐r2C*, respectively. EV adoption under the current energy generation mix leads to PM‐related reduced annual mortality that decreases by 437 (95% CI: 295, 578) in *e25‐rC* to 1,576 (95% CI: 1065, 2086) in *e75‐rC* and O_3_‐related annual mortality that decreases by 98 (95% CI: 33, 162) to 420 (95% CI: 139, 698), respectively. These reported reduced mortality estimates are obtained from the most conservative dose‐response functions we employ (i.e., Bell et al., 2004; Krewski et al., 2009), and comparison with other HIFs (see Table 3 and section 3.3) suggests that the magnitude of avoided mortality could double our estimates.

The largest magnitude national‐level public health and climate change co‐benefits occur in our aggressive adoption scenario when added energy demand for charging is predominantly supplied by emission‐free energy sources (*e75‐r2C*). However, in the scenario with the same EV proportion but status‐quo energy mix (*e75‐rC*), the magnitude of CO_2_ decrease is slight (~9%), while PM_2.5_‐related health benefits decrease ~46%. The disproportionate influence of the energy mix composition on PM_2.5_ has implications for disparate regional health outcomes of vehicle electrification, which we explore further in section 3.2.

### 3.3 Region‐ and State‐Level Co‐benefit/Trade‐Off Patterns

#### 3.3.1 CO_2_ Emission Changes

While we find that aggregate U.S. CO_2_ emissions are reduced in all simulated EV scenarios, at the state level there is one exception (Figures 2b and 2e; Table S2). Under the combustion‐only (*r0*) energy generation scenarios, Nebraska experiences CO_2_ emission increases of 0.06 Mt year^−1^ in *e25‐r0* and 0.18 Mt year^−1^ in *e75‐r0* due to a combination of fewer LDPV emissions, high CO_2_ emissions from EGUs, and the charging demand of neighboring states. Notably, CO_2_ emissions associated with EV charging are tallied where they are produced (i.e., at the power station) and are not necessarily associated with EV charging in that state; that is, a state can see an increase in CO_2_ emissions from out‐of‐state vehicle charging. Indeed, Nebraska experiences CO_2_ reductions of 0.68 and 2.0 Mt year^−1^ respectively for *e25‐r0* and *e75‐r0* if only EGU emissions from Nebraska's charging demand is considered. CO_2_ mitigation increases moderately with added emission‐free energy generation (*r0* → *rC* → *r2C*), but the total reduction in CO_2_ is primarily driven by the replacement of fossil fuel vehicle miles with higher‐efficiency energy from power generation stations. For example, California receives the largest CO_2_ emission reduction for all EV scenarios, and the statewide change from *e25‐r0* to *e25‐r2C* reduces CO_2_ emissions by ~3 M tons, whereas the increased EV proportion from *e25‐r0* to *e75‐r0* tripled the CO_2_ emission reduction from 29 to 88 M tons per year.

#### 3.3.2 Health Benefits and Trade‐Offs From Ozone Changes

**Figure 3:**
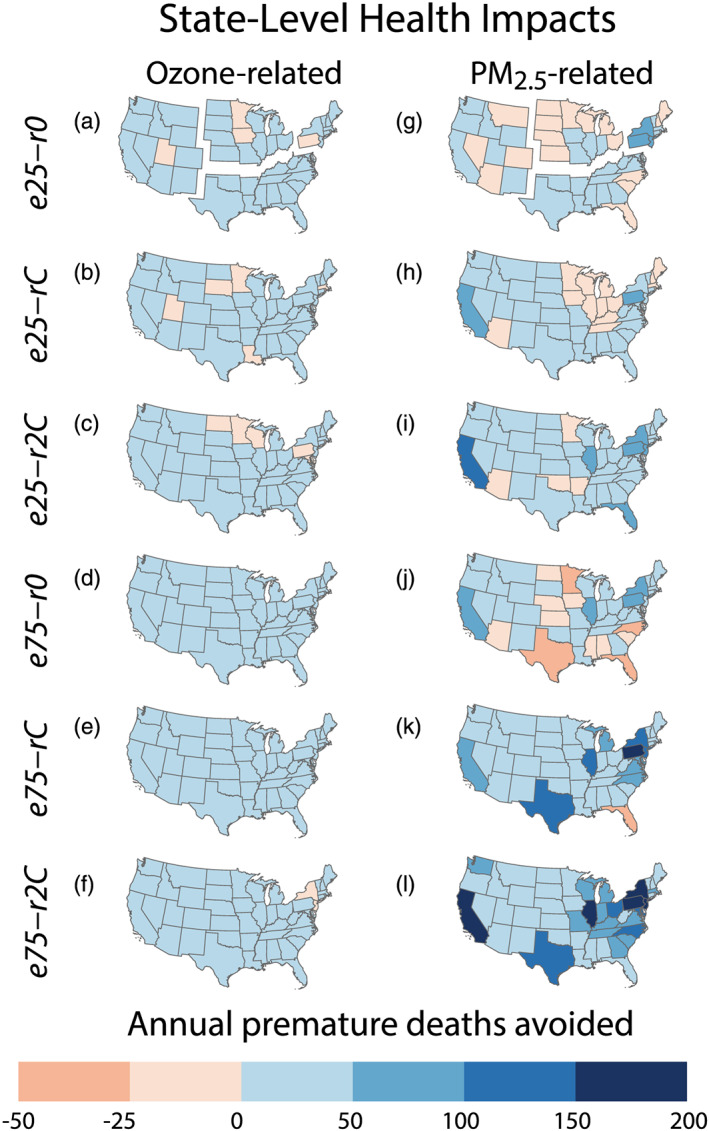
Annual premature deaths avoided. EV adoption scenario‐driven changes in air pollutants (a–f) O_3_ (Bell et al., 2004) and (g–l) PM_2.5_ (Krewski et al., 2009) drive changes in annual premature mortality incidence. Negative numbers signify increases in premature mortality. Panels (a) and (g) are subdivided into U.S. Census regions: Midwest, West, northeast, and south (U.S. Census Bureau, 2018). For population normalized state and grid cell level data see Figures S1 and S2.

Ozone health benefit patterns are similar to CO_2_ mitigation benefits, in that they are largely consistent across the six electrification scenarios (Figure 2a). Health benefits from O_3_ reduction in individual states in all scenarios are generally positive, relatively modest, and do not exceed 50 fewer premature deaths per year in any locale (Figures 3a–3f; for population normalized data see Figures S1a–S1f and S2a–S2f)). EV scenarios with combustion‐only energy generation still yield O_3_ health benefits in most states (Figures 3a and 3d). Under the *e25‐r0* scenario, wherein EV charging demand is met by combustion‐only power generation sources, O_3_ decreases, and attendant health benefits are nearly pervasive, however increases in premature mortality are simulated in UT, IA, MN, and PA (Figure 3a). Under the *e75‐r0* scenario, however, the O_3_ decreases and health benefits occur over all states (Figure 3d). Notably, in *r2C* scenarios with doubled statewide emission‐free power generation, isolated states see increased O_3_‐driven premature deaths. In *e75‐r2C* the states of New York and New Jersey by and large see O_3_ reductions, but O_3_ increases in VOC‐limited urban grid cells nullify gains in the majority of each state (Figure S2f). Increases in state‐wide premature deaths are minute (NJ: 2.6 year^−1^ and NY: 0.1 year^−1^), yet positive (Figures S1f and S2f).

**Figure 4:**
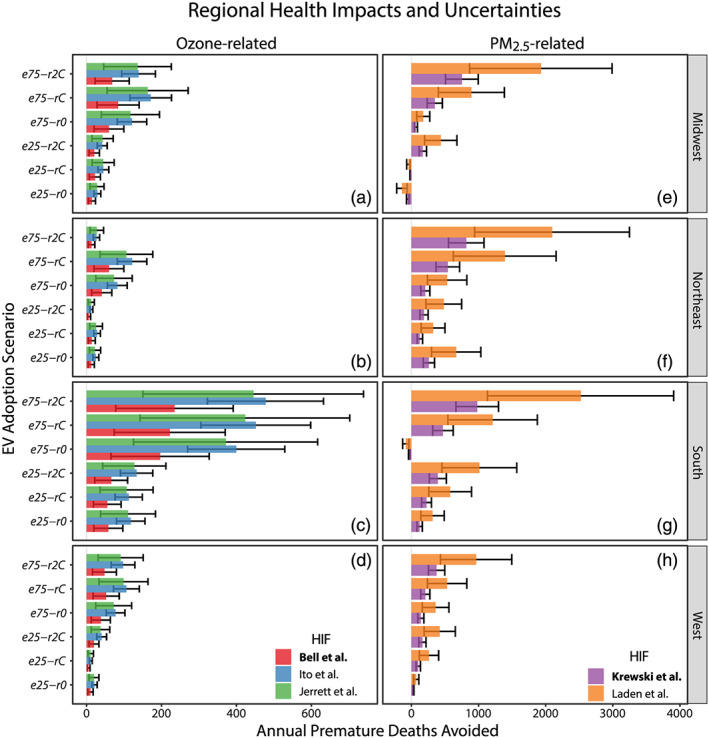
Regional health outcome uncertainties. Regional premature mortality changes under different EV adoption scenarios and different HIFs. Error bars reflect 95% CI of HIFs. See Figure 3 for regional U.S. Census demarcations.

By region, the South experiences the largest absolute magnitude of O_3_‐related health benefits (Figure 4). For states in the South, median deaths avoided per year range from 56 to 235 across the adoption scenarios (red bars; Figure 4c). In other regions, health benefits due to O_3_ reductions are more modest, that is, in all scenarios and all non‐South regions cumulative reductions in premature mortality fall below 100 per year. Our finding that the largest positive health changes are driven by LDPV replacement fraction holds true across regions. Regional median premature deaths avoided per year increase between the *e25‐rC* and *e75‐rC* scenarios from 6 to 42 in the West, 56 to 222 in the South, 14 to 60 in the Northeast, and 22 to 84 in the Midwest. Curiously, we find that in the West, Midwest, and Northeast regions premature mortality reductions decrease as the fraction of emission‐free power generation increases, that is, annual deaths avoided in *rC* scenarios are greater than in *r2C* scenarios (Figure 4). This counterintuitive result is driven by nonlinear O_3_ chemistry in the high population, high emission urban cores of these regions; that is, with sufficient NO_x_ reductions, O_3_ production efficiency increases (Sillman, 1995).

#### 3.3.3 Health Benefits and Trade‐Offs From PM_2.5_ Changes

Unlike CO_2_ and O_3_ outcomes, the distribution of PM_2.5_‐related health consequences is spatially variable and shifts substantially between electrification scenarios (Figures 2b–2g and 3g–3l). In an absolute sense, the most‐substantial PM_2.5_ health benefits under most scenarios are found in California, Illinois, and the northeastern states of New York, New Jersey, and Pennsylvania (Figure 3). Population normalized health outcomes suggest a somewhat similar pattern; however on a per capita basis the Midwest and mid‐Atlantic states see the largest benefits with emission‐free power usage (Figures S1g–S1l, S2g–S2l, and S3a–S3f). Health outcomes for EV adoption under current energy generation infrastructure vary according to fraction of internal combustion engine replacement. The *e25‐rC* simulation reduces PM_2.5_‐related premature mortality in most states, with the exception of the Midwest, Arizona, and New England (Figure 3h). Under the *e75‐rC* scenario, however, all but one state (FL) sees reduced premature mortality (Figure 3k).

When EV adoption uses combustion‐only power generation, health outcomes are decidedly more mixed (Figures 3g and 3j). Some northeastern states (e.g., New York and Pennsylvania) consistently experience PM_2.5_‐related benefits even under combustion‐only charging scenarios due to significant reductions in urban traffic PM_2.5_ emissions and precursors and fewer coal‐fired power plants in their grid mix. Under *e75‐r0*, we find that 12 states, located mostly in the South and Midwest, experience increases in PM_2.5_‐related mortality. This finding is consistent with simulated PM_2.5_ increases that result from increases in ammonium sulfate emissions in the summer, driven by SO_2_ emissions from coal‐fired power plants (Schnell et al., 2019). Texas, Florida, North Carolina, and Minnesota experience a combined simulated increase of 132 deaths in the *e75‐r0* scenario (Figure 4j). By contrast, when the same proportion of EVs is charged by higher levels of emission‐free energy generation sources (*e75‐r2C*), these four states experience PM_2.5_ health benefits totaling 330 avoided premature deaths per year (Figure 4l). Indeed, when aggressive EV adoption is paired with expanded emission‐free power generation (*e75‐r2C*) all states see substantial reductions in annual PM_2.5_‐related premature mortality.

On a regional basis, we find that all sectors have the potential for substantial PM_2.5_‐related public health improvements (Figures 2b–2g and 4). The West and Northeast experience net positive benefits of avoided mortality across all scenarios. The Northeast sees the greatest PM_2.5_ health benefits of any region in combustion‐only (*eX‐r0*) simulations (Figure 4f) and has larger benefits for *e25‐r0* than *e25‐rC*, a result of nonlinear NO_x_ chemistry to generate nitrate aerosol (Figure S10 in Schnell et al., 2019). Under electrification scenarios with doubled fractions of emission‐free power generation (*eX‐r2C*), PM_2.5_ health benefits in the South exceed other regions, with 394 (95% CI: 266, 521) premature deaths avoided per year in the *e25‐r2C* and 981 (95% CI: 662, 1,298) in *e75‐r2C* (Figure 4g). At 75% EV penetration, the South also has the greatest range of benefits between power generation scenarios; from a predicted increase of 32 premature deaths per year with combustion‐only energy sources (*e75‐r0*) to the decrease of 981 deaths per year with doubled emission‐free energy sources (*e75‐r2C*). The Midwest also has aggregate increases in adverse health effects under two of the 25% EV penetration scenarios (*e25‐r0* and *e25‐rC*) with 55 and 18 additional premature deaths per year predicted under these respective scenarios. The PM_2.5_ scenario in which each region experiences peak avoided mortality is the most ambitious adoption and electrification scenario (*e75‐r2C*; Figures 2b–2g; Figure S3).

### 3.4 Health Impact Uncertainties

The above reported and discussed health outcomes are based on HIFs from Bell et al. (2004) for O_3_ exposure and Krewski et al. (2009) for PM_2.5_ exposure. However, we also applied HIFs from Ito et al. (2005) and Jerrett et al. (2009) for O_3_ and Laden et al. (2006) for PM_2.5_ for all EV adoption‐energy generation scenarios considered (Figure 4). Our chosen focus on Bell et al. and Krewski et al. is rooted in their relatively conservative predictions in comparison to the other HIFs considered. However, to elucidate uncertainties in HIFs, for example, differences in mathematical formulation, demographics considered, and temporal exposure (Table S1), we provide national, regional, and state‐level comparisons between all HIFs for both O_3_ and PM_2.5_ exposure (Table 3, Figure 4, Tables S3–S5). For both modeled pollutants, the estimated health impacts vary substantially between HIFs. At the aggregate national level for PM_2.5_, the Laden et al. HIF estimates health benefits that are consistently more than double Krewski et al. (Figures 4e–4h). Krewski et al.'s HIF predicts 358 (95% CI: 242, 473) premature deaths avoided per year under the least ambitious scenario (*e25‐r0*) and 2,939 (95% CI: 1,985, 3,888) for the most ambitious (*e75‐r2C*), whereas Laden et al.'s function predicts between 919 (95% CI: 412, 1,423) and 7,548 (95% CI: 3,386, 11,684) deaths avoided (Table 3). We also tested the sensitivity of our PM_2.5_ health impact results to a third function, Lepeule et al. (2012), which was a follow‐up analysis to the Harvard Six Cities study that included data through 2009, that is, an 11‐year follow‐up on Laden et al. (2006). The Lepeule et al. HIF results were similar to our Laden et al. analysis (i.e., 7,548 vs. 6,665 deaths avoided per year for *e75‐r2C*), while much greater than the 2,939 deaths avoided per year predicted by the Krewski et al. HIF. For O_3_, the Ito et al. and Jerrett et al. functions yield similar estimates for each scenario, though Ito et al. has lesser uncertainties, while Bell et al. HIF estimates are generally about half the magnitude of the other two O_3_ exposure functions. The three O_3_ functions predict between 96 (95% CI: 32, 159) and 193 (95% CI: 131, 255) annual premature deaths avoided nationally under the *e25‐r0* scenario and up to 366 (95% CI: 121, 608) to 742 (95% CI: 501, 982) premature deaths avoided under the *e75‐r2C* scenario (Table 3).

## 4 Discussion and Conclusion

Our analysis of six EV adoption‐energy generation scenarios indicates that vehicle electrification in the United States could annually prevent hundreds‐to‐thousands of premature deaths while also reducing CO_2_ emissions by hundreds of millions of tons. Estimates of economic damages avoided due to EV adoption are substantial. With current infrastructure and 25% EV adoption (*e25‐rC*) we find savings of $16.8B annually (i.e., $11.6B U.S. SCC and $5.1B VSL). In more aggressive scenarios, for example, *e75‐r2C*, savings of $70B year^−1^ are found (i.e., $38.2B U.S. SCC and $31.7B VSL). However, we also find that PM_2.5_ changes and corresponding health impacts vary across U.S. regions, and the realization of co‐benefits for PM_2.5_ depends largely on the energy sources used to charge EVs. The scenarios that we assess, which assume instantaneous EV replacement of conventional LDV's, are not intended to simulate dynamic real‐world EV uptake, but rather serve as sensitivity tests to estimate the magnitude and geographic distribution of mitigation outcomes under varied energy regimes and EV replacement proportions. Additionally, our health impact estimates of avoided mortality and the corresponding economic valuations should not be seen as comprehensive because (a) there is well‐documented evidence of a variety of non‐mortality health outcomes associated with transportation‐related air pollution, including asthma and other cardio‐pulmonary ailments, and (b) our study only considers ground‐level O_3_ and PM_2.5_ exposure, while there may be additional impacts from changes in direct exposure to NO_2_ and other transport emissions (Anenberg et al., 2018).

Simulated health impact results are influenced by a number of limitations and uncertainties, including HIF cohorts and assumptions, structural chemistry‐climate model biases, meteorological variability, and population trends. Our sensitivity analysis using several HIFs demonstrates that relative risk (RR) estimates, and corresponding premature mortality estimates, can vary substantially between epidemiological studies. Our application of HIFs also assumes that the results of cohort studies with specific geographies and populations (Table S1) can be applied nationwide where factors including population demographics, environmental characteristics, and pollutant concentrations will vary from the cohort study conditions. Likewise, the structural formulation of individual chemistry‐climate models leads to inherent model response uncertainties, which could ideally be addressed using a multimodel assessment framework (Hawkins & Sutton, 2009). Furthermore, computational demands have limited simulations to a single historic year and therefore do not consider EV adoption and health outcomes in the context of internal climatic variability, a limitation that could be addressed with initial‐condition perturbation experiments (Garcia‐Menendez et al., 2017). The temporal context of the experiment also presents uncertainties regarding applicability to future years. The meteorology and level of radiative forcing of the modeled year influence pollutant formation, and therefore should be considered when attempting to project future outcomes and applicability (Fiore et al., 2015). Lastly, health impact estimates depend on population demographics and baseline mortality incidence rates, which are expected to shift over time.

National CO_2_ mitigation estimates follow a pattern consistent with previous studies that have shown EVs to significantly reduce CO_2_ emissions even when the charging energy is sourced from carbon‐intensive energy generation facilities including coal combustion (Requia et al., 2018). The range of CO_2_ mitigation estimates from our six scenarios represents approximately 18 to 66% of the total 2014 CO_2_ emissions attributed to light‐duty vehicles in the U.S.‐48 (U.S. EPA, 2014). We find that even if the efficiency of the assumed EV was decreased by 30% (i.e., increasing the electricity demand by 30%), CO_2_ reductions are still widely apparent—ranging from 192 to 767 Mt year^−1^. While these results do not account for the life cycle emissions of EV battery production and disposal, based on past studies we would expect inclusion of battery production emissions to reduce CO_2_ mitigation benefits by 5% or less (Samaras & Meisterling, 2008). Further, rapid advances in battery technology, low‐carbon manufacturing processes, and the greening of the grid suggest that estimates provided here are likely conservative and that purchased EVs will get cleaner over their lifetimes.

The magnitude of simulated health consequences from PM_2.5_ reductions far exceeds the benefits from O_3_ changes for most vehicle electrification scenarios. Indeed, the O_3_‐attributed national aggregate benefits from our most extreme hypothetical with 75% EV conversion and double emission‐free charging (*e75‐r2C*) are comparable with the PM_2.5_‐related benefits under the least ambitious of our EV scenarios (*e25‐r0*). The difference in magnitude between PM_2.5_ and O_3_‐related benefits in our experiments is consistent with literature that indicates the higher relative mortality burden of mobile‐source PM_2.5_ in the United States compared to O_3_ and reflects the higher risk coefficient associated with PM_2.5_ exposure compared to O_3_ exposure (Fann et al., 2013).

The overall magnitude of PM_2.5_ health impact estimates is consistent with most of the limited available literature. Grabow et al. (2012) modeled the health impacts of reducing residential car travel by 20% in urban areas throughout the Midwest and found that the corresponding PM_2.5_ reductions would reduce mortality by 525 deaths per year. This result is similar in magnitude to our estimate of 170 (Krewski et al. HIF) to 437 (Laden et al. HIF) avoided mortalities for the Midwest region under the *e25‐r2C* scenario that is most comparable to Grabow et al.'s experiment. Indeed, we would expect our values to be more modest in comparison, as the former study fully eliminated 20% of residential car trip emissions, whereas our simulations reflect the transfer of tailpipe emissions to powerplant emissions. Furthermore, Jacobson et al. (2005) modeled the mortality impacts of 100% instantaneous replacement of fossil fuel on‐road vehicles with hydrogen vehicles, where the hydrogen was produced through 100% wind electrolysis, finding that PM_2.5_ changes avoided 3,710–6,350 deaths per year. For the *e75‐r2C* scenario we estimate a range of 2,939 (Krewski et al. HIF) to 7,548 (Laden et al. HIF) deaths avoided per year, which is a similar magnitude to Jacobson et al.'s finding under a comparable scenario both in EV proportion (75%) and in the high levels of renewable energy used to power the alternative fuel vehicles.

Our results demonstrate that PM_2.5_ health benefits are not guaranteed under high EV penetration scenarios in some U.S. regions (e.g., Midwest and South), and emission‐free energy sources can be the difference between positive and negative state‐level health outcomes of vehicle electrification. Given that EV air quality studies (e.g., Ji et al., 2015) have predicted a shift in air pollution burden from urban to rural areas with vehicle electrification—the so‐called spillover effect (Fang et al., 2019)—future analyses, ideally employing a higher spatial resolution modeling framework, should investigate the public health consequences of the geographic redistribution of air pollution, which may be an important environmental justice consideration for electric vehicle policy decisions (Ji et al., 2015). This is especially true for urban cores, where the chemical regime (i.e., NO_x_‐ vs. VOC‐limited) and population density may differ substantially over a few tens of kilometers, all of which would be averaged over a single ~50 km grid cell in this analysis. Similarly, analyses seeking to project policy outcomes, such as EV adoption, in a future world should strive to characterize all forms of projection uncertainty, that is, internal variability, scenario, and model structure uncertainties, and their implications on projected outcomes (Deser et al., 2020; Kinney, 2008). Studies have projected significant future emissions reductions due to fleet turnover and existing policies such as vehicle fuel and emission standards (Davidson et al., 2020); future work comparing co‐benefits from such regulations to vehicle electrification could give insight into the most effective levers to maximize progress toward cleaner air and reduced GHG emissions.

Reducing the environmental burden from the transportation sector is a compelling opportunity to address the two global challenges of air pollution and climate change. In this study, we show the potential distribution of health consequences from vehicle electrification scenarios in the United States and compare the climate CO_2_ mitigation outcomes with the health benefits and trade‐offs. We find that while U.S. vehicle electrification is expected to significantly reduce transportation CO_2_ emissions and has the potential to improve air quality and mitigate thousands of annual premature deaths, the extent and magnitude of health co‐benefits largely depend on the charging energy mix, particularly for changes in PM_2.5_. The results show that while electric vehicles under status quo energy regimes produce significant CO_2_ reductions, the greatest health co‐benefits are achieved by electrifying vehicles and charging with a greater fraction of emission‐free electricity generation sources. This finding is consistent with Tessum et al. (2014), who demonstrates the importance of coincident fleet electrification and grid decarbonization to achieve maximum co‐benefits. We add to this understanding by elucidating co‐benefits and trade‐offs on a state‐by‐state basis and by altering the EV replacement proportion to demonstrate the impact on the magnitude and distribution of climate and health outcomes. Our results reinforce the importance of assessing air quality and health consequences in relation to EV adoption goals. When decision‐makers pursue vehicle electrification as a strategy for carbon emission reduction, our results indicate opportunity for widespread and significant public health benefits, but they also show the potential regional trade‐offs when EVs are charged by combustion energy generation sources. Policymakers should favor a comprehensive climate action plan that ensures the public will experience optimal health co‐benefits in addition to the CO_2_ reductions associated with vehicle electrification.

## Conflict of Interest

The authors declare no conflicts of interest relevant to this study.

## Supporting information

Supporting Information S1

Table S1

Table S2

Table S3

Table S4

## Data Availability

Simulations were performed using the Geophysical Fluid Dynamics Laboratory Atmospheric Model, version 4 (GFDL AM4; Zhao et al., 2018a, 2018b). Experimental configuration of the simulations presented here are reported in the Methods section and in Schnell et al. (2019).
