# Supplementary material for: Public Health and Climate Benefits and Trade‐Offs of U.S. Vehicle Electrification
Source: Geohealth. 2020 Oct 1;4(10):e2020GH000275. doi: 10.1029/2020GH000275 (PMC7567144; doi:10.1029/2020GH000275)
Supplement: Supplementary file 1 — Supporting Information S1 [file GH2-4-e2020GH000275-s001.pdf]

AGU PUBLICATIONS

GeoHealth

Supporting Information for

Public Health and Climate Benefits and Tradeoffs of U.S. Vehicle Electrification

D. R. Peters[1,2], J. L. Schnell[3,4], P. L. Kinney[5], V. Naik[6], & D. E. Horton[3]

[1]Program in Environmental Sciences, Northwestern University
[2]Environmental Defense Fund, Austin, TX
[3]Department of Earth and Planetary Sciences and Institute for Sustainability and Energy at Northwestern University
[4]Cooperative Institute for Research in Environmental Sciences at the University of Colorado Boulder NOAA/Global Systems Laboratory, Boulder, Colorado, USA
[5]Department of Environmental Health, Boston University School of Public Health
[6]NOAA Geophysical Fluid Dynamics Laboratory, Princeton, NJ

Contents of this file

Figures S1 to S3
Table S1

Additional Supporting Information (Files uploaded separately)

Captions for Tables S2 to S5

Introduction

The supporting information contains three figures, which expand on the main text figures by displaying health impact co-benefit results on a per-capita scale. Additionally, the supporting information includes five tables which contain the calculated state-level CO2 emissions changes, the BenMAP-CE health impact function parameters, and the BenMAP-CE health impact function results aggregated nationally and by U.S. state and region.

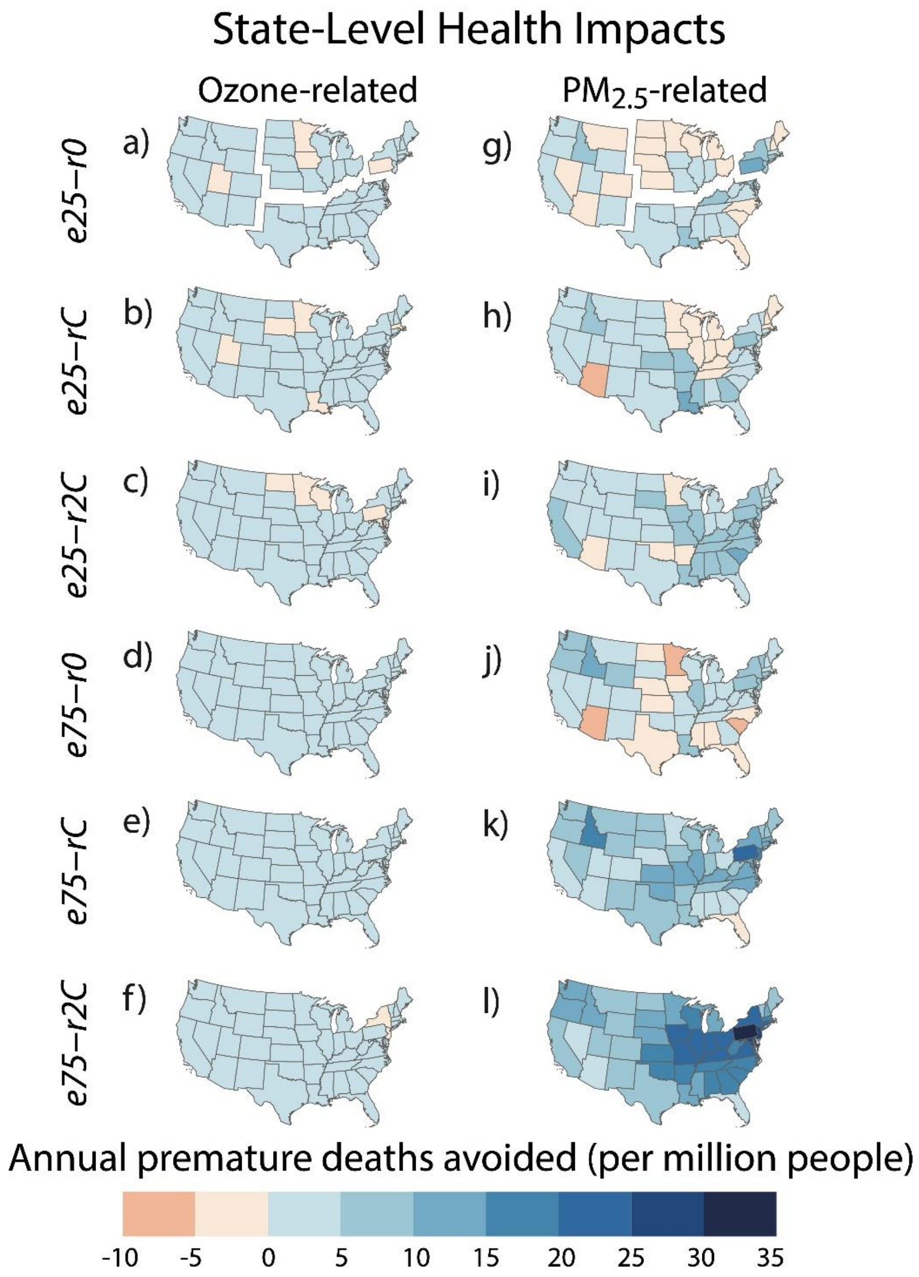

Figure S1. Population normalized annual premature deaths avoided by state. EV adoption scenario-driven changes in air pollutants – (a-f) O_3 (Bell et al., 2004) and (g-l) $PM_{2.5}$. (Krewski et al., 2009) – drive changes in annual premature mortality incidence. Data is normalized by state population. Negative numbers signify increases in premature mortality. Panels a and g are subdivided into U.S. Census regions; Midwest, West, Northeast, and South (U.S. Census Bureau, 2018).

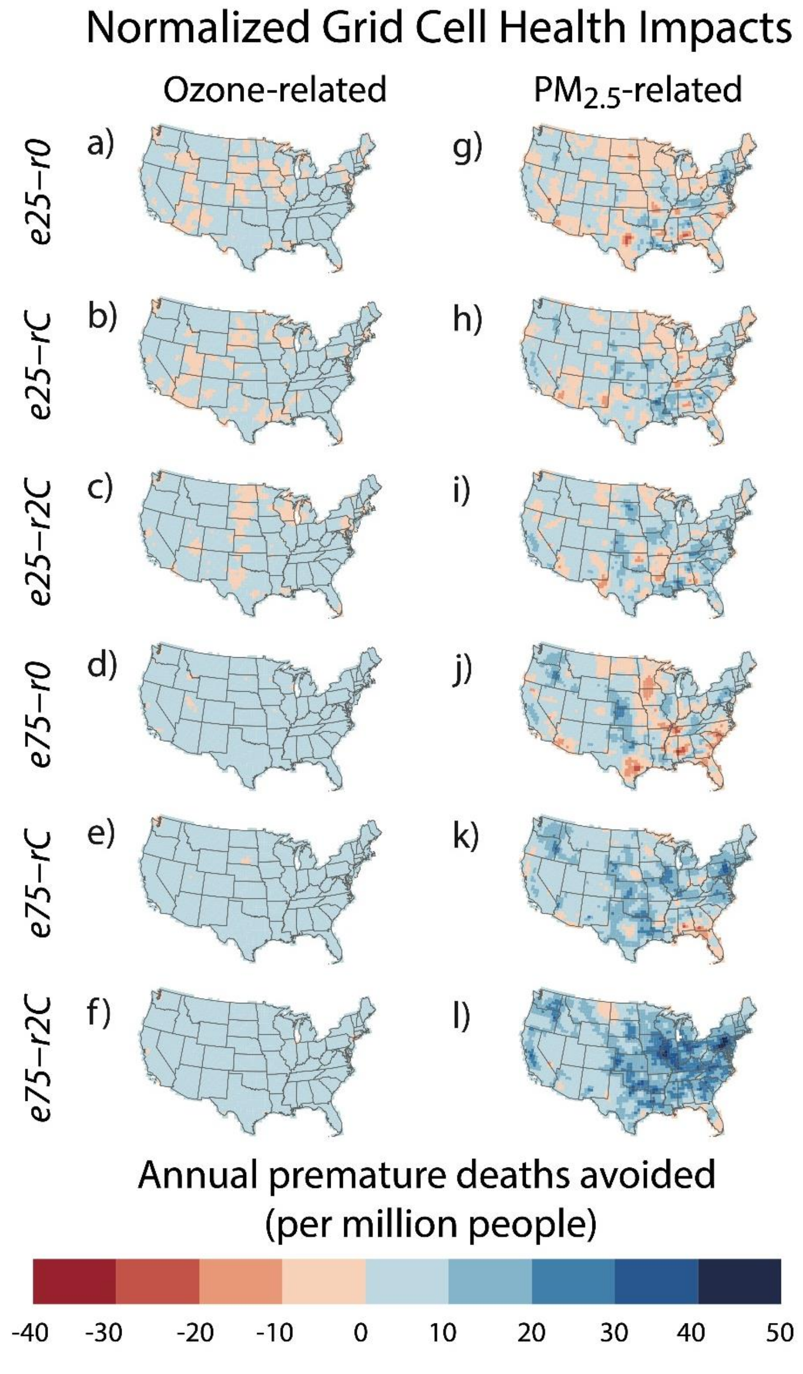

Figure S2. Population normalized annual premature deaths avoided by model grid cell. EV adoption scenario-driven changes in air pollutants – (a-f) O_3 (Bell et al., 2004) and (g-l) PM2.5. (Krewski et al., 2009) – drive changes in annual premature mortality incidence. Data is normalized by grid cell population. Negative numbers signify increases in premature mortality.

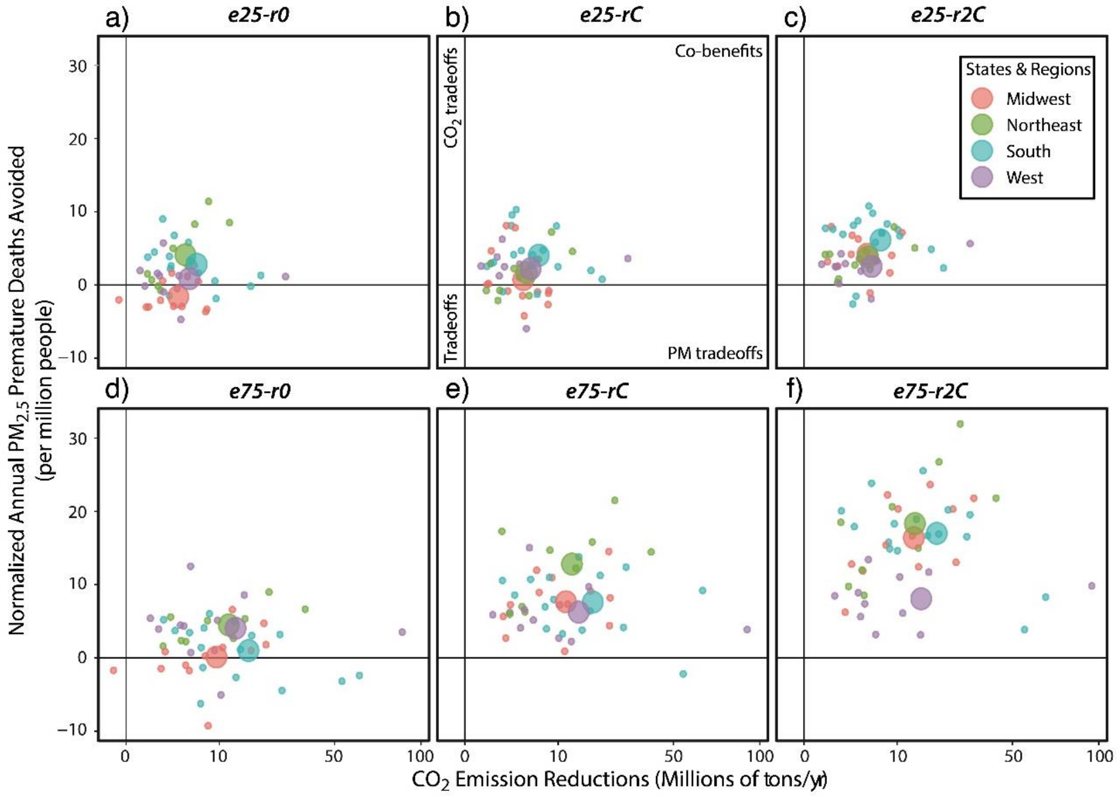

Figure S3. Population normalized national, regional, and state co-benefits. Avoided premature mortality and CO_2 reduction co-benefits under six vehicle electrification scenarios. Climate and $PM_{2.5}$ health co-benefits (Krewski et al., 2009) for individual states (smaller circles) and regional averages (larger circles) normalized by population.

Health Impact Function (HIF)	Pollutant	Location	Age Group	Health Endpoint	Concentration-response coefficient (β)	β Standard Error	Form
Krewski et al.	$PM_{2.5}$ (Annual)	116 U.S. Cities	30-99	All-Cause Mortality (Long-Term)	0.005827	0.000963	Log-linear
Laden et al.	$PM_{2.5}$ (Annual)	6 Cities	25-99	All-Cause Mortality (Long-Term)	0.014842	0.004170	Log-linear
Bell et al.	Ozone (MDA8)	95 U.S. Cities	0-99	Non-Accidental Mortality (Short-Term)	0.0002613	0.000089	Log-linear
Ito et al.	Ozone (MDA8)	7 U.S Cities	0-99	Non-Accidental Mortality (Short-Term)	0.000532	0.000088	Log-linear
Jerrett et al.	Ozone (Annual)	86 U.S. Urban Areas	30-99	Respiratory Mortality (Long-Term)	0.004471	0.001510	Log-linear

Table S1. Source of health impact functions (HIFs) and underlying characteristics (US EPA, 2019b).

Tables S2 – S5 are provided as .csv files.

Table S2. State-level changes in CO_2 emissions (10^6 tons per year) for EV adoption-energy generation scenarios.

Table S3. National health impact data. Column 1: scenario (1 = *e25r0*; 2 = *e25rC*; 3 = *e25r2C*; 4 = *e75r0*; 5 = *e75rC*; 6 = *e75r2C*). Column 2: O_3 or $PM_{2.5}$ pollutant. Column 3: HIF Author (see supplemental Table 1 for more details). Column 4, 5, & 6: 50P, 2.5P, and 97.5P estimates of avoided mortality, respectively.

Table S4. Regional health impacts data. Column 1: scenario (1 = e25r0; 2 = e25rC; 3 = e25r2C; 4 = e75r0; 5 = e75rC; 6 = e75r2C). Column 2: O3 or PM2.5 pollutant. Column 3: HIF Author (see supplemental Table 1 for more details). Columns 4, 5, & 6: 50P, 2.5P, and 97.5P estimates of avoided mortality, respectively.

Table S5. State health impact data. Column 1: state. Column 2: scenario (1 = *e25r0*; 2 = *e25rC*; 3 = *e25r2C*; 4 = *e75r0*; 5 = *e75rC*; 6 = *e75r2C*). Column 3: HIF Author (see supplemental Table 1 for more details). Column 4: state population. Columns 5, 6, & 7: 50P, 2.5P, and 97.5P estimates of avoided mortality, respectively. Column 8: percent of baseline; HIF 50P estimate of annual avoided mortality normalized by baseline (*BASE*) incidence rates. Column 9: region of U.S. based on Census Bureau Classification (2018). Column 10: O_3 or $PM_{2.5}$ pollutant.
